# Pilot cluster randomised controlled trial of Stepping Stones and Creating Futures Plus to reduce emotional dysregulation among young men in rural areas and urban informal settlements in KwaZulu-Natal Province, South Africa

**DOI:** 10.1177/00207640241306062

**Published:** 2024-12-26

**Authors:** Princess Nyoni, Andrew Tomita, Smanga Mkhwanazi, Andrew Gibbs

**Affiliations:** 1 School of Nursing and Public Health, Discipline of Public Health Medicine, University of KwaZulu-Natal, Durban, South Africa; 2 Health Economics and HIV and AIDS Research Division, University of KwaZulu-Natal, Durban, South Africa; 3 Centre for Rural Health, School of Nursing and Public Health, University of KwaZulu-Natal, Durban, South Africa; 4 KwaZulu-Natal Research Innovation and Sequencing Platform, College of Health Sciences, University of KwaZulu-Natal, Durban, South Africa; 5 Gender and Health Research Unit, South African Medical Research Council, Pretoria, South Africa; 6 Department of Psychology, University of Exeter, UK

**Keywords:** Emotional dysregulation, mental health, SSCF+ intervention, men, South Africa

## Abstract

**Aim:**

Emotional dysregulation (ED) – the difficulty to control emotional responses to stressors – is a potential driver of intimate partner violence (IPV) perpetration among young men in HIV endemic resource-limited settings. This two-armed pilot cluster randomised controlled trial investigated the effectiveness of Stepping Stones and Creating Futures Plus (SSCF+), a participatory gender transformative and livelihood strengthening intervention, on the emotional dysregulation (ED) among young men in South Africa (SA).

**Methods:**

A total of 163 young men ages 18 to 30 years were recruited in 30 clusters (friendship groups) in urban informal settlements and rural areas in KwaZulu-Natal, SA. Clusters were randomly allocated (1:1) to either the experimental SSCF+ or control arm, stratified urban/rural and participants were followed-up at 5 months. Intention-to-treat analysis based on generalised estimating equations (GEE) were fitted to quantify the impact of SSCF+ on the men’s ED using the culturally tested short version of the Difficulties in Emotion Regulation Scale with 16 items (DERS-16).

**Results:**

At 5 months SSCF+ did not significantly reduce ED in the overall sample. However, SSCF+ had a significant impact on ED among the men at risk of depression at baseline (adjusted odds ratio = 0.12, 95% CI [0.03, 0.46], *p* = .002).

**Conclusion:**

SSCF+, a gender transformative and livelihoods strengthening intervention designed to address poverty and other socio-economic challenges, reduced ED among youth with depression challenges in KwaZulu-Natal Province, South Africa.

## Introduction

Intimate partner violence (IPV) is a pressing public health problem in South Africa (SA; Mthembu et al., 2021). Women typically experience IPV (Gass et al., 2011) and those who experience IPV are also more likely to acquire HIV (Jewkes et al., 2010), a particular concern given the continued high rates of HIV-acquisition among young women (15–25 years) and the young people in general (South Africa National AIDS Council, 2022; Zuma et al., 2022). The impact of the synergistic clustering of IPV and HIV among young people is often most pronounced in situations with environmental and economic adversities, such as rural areas and urban informal settlements in South Africa. Both settings have limited economic activities and lack basic services such as clean water and sewage system (du Toit, 2017; Weimann & Oni, 2019; Wilna & Slabbert, 2010). High rates of poverty indicators such as food insecurity, have also been reported, one rural study reported two-thirds (67.4%) of inhabitants were food insecure (Tambe et al., 2023). Another study conducted in an urban informal settlement reported more than 60% being food insecure (Mkhize & Sibanda, 2022). The multiple hardships associated with living in urban informal settlements and rural areas could explain why some research has identified high IPV rates in these areas of 65.2% (Gibbs et al., 2018) and up to 92.2% (Allen, 2017), as well as the high risk of acquiring HIV (Gibbs et al., 2020; Linganiso & Jmt, 2016).

The adversities young men face in rural areas and urban informal settlements may increase their risk of poor mental health. A study conducted by Oyekunle et al. (2023) found high rates of poor mental health (i.e. prevalence of depression and post-traumatic stress being 46.8% and 14.4% respectively) among young (18–30 years) men in urban informal settlements in SA. There is strong evidence that men who have mental health disorders such as, post-traumatic stress (PTS) and depression are more likely to perpetrate violence (Machisa et al., 2016). In a retrospective cohort of Swedish men based on nationwide registries, men with mental health disorders (e.g. depression) were most likely to perpetrate violence against women compared to the controls and their findings were adjusted for possible confounders (Yu et al., 2019). Another study conducted in Zimbabwe with a nationally representative sample showed depressive symptoms and PTS to be associated with perpetration of violence whilst controlling for possible confounders (M. Machisa & Shamu, 2018). A meta-analysis which included 207 studies from various countries also found PTS, depression and anxiety to be associated with IPV perpetration (Spencer et al., 2019).

Emotional dysregulation (ED), sometimes referred to as a trans-diagnostic symptom of several mental health disorders (Carmassi et al., 2022), has not been studied in urban informal settlements and rural areas in SA, yet it may also contribute to both IPV perpetration and HIV acquisition risk. Studies have shown that ED is associated with violence perpetration in studies conducted in Sri Lanka (Bliton et al., 2016) and in USA (Conzemius et al., 2021). Additionally, studies in Iran found that poor emotion regulation increases the risk of engaging in risky sexual behaviours (Hashemi et al., 2018; Manee, 2014), which are a key risk factor for acquiring HIV.

ED can be defined as the inability to regulate emotions healthily that is an emotional response that does not fall within the traditionally accepted range (Tacchini & Vismara, 2019), such as uncontrollable anger and destroying things. ED has various dimensions, including lack of emotional clarity and awareness, inability to engage in goal-oriented behaviours, difficulty controlling impulse behaviours when distressed, lack of confidence in or limited access to effective emotion regulation strategies and none-acceptance of negative emotions (Gratz & Roemer, 2004).

A range of individual and social factors have been associated with ED. A common risk factor for ED is the presence of existing mental health disorders. Studies conducted in USA and Europe found those with major mental health disorders that is anxiety, PTS and depression to struggle to regulate their emotions (Etkin et al., 2010; Shepherd & Wild, 2014; Visted et al., 2018). Poverty is also associated with increased risk of emotional dysregulation, with studies in USA finding this relationship among children (Liberzon et al., 2015). More widely, reviews have found a significant relationship between poverty (food insecurity) and poorer mental health in individuals (Ejiohuo et al., 2024). Finally, older age has been found to be protective of ED in studies conducted in England (Brummer et al., 2013; Orgeta, 2009) and Japan (Nakagawa et al., 2017).

Given the large burden of IPV, HIV and poor mental health in rural areas and urban informal settlements in SA especially among young people, interventions have increasingly been designed and implemented to try and address these. One intervention that was designed to address some of these challenges faced by the young people in these contexts is the Stepping Stone and Creating Futures Plus (SSCF+) intervention, adapted and modified from the Stepping Stone and Creating Futures intervention (Gibbs et al., 2017). SSCF+ sought to address the overlapping challenges of poor mental health, IPV and poverty, through addressing gender inequality, improving the ability to relate positively with others, encouraging the developing of livelihoods and supporting improved wellbeing. It differed from SSCF in several ways, including a much stronger focus on addressing poor mental health through inclusion of components from Self-Help Plus and Problem Management Plus (World Health Organization, 2021), it was delivered to small friendship groups, rather than larger groups, and was substantially shorter as an intervention. It was co-developed with young people in these contexts of hardships (i.e. one rural area and urban informal settlements), and practitioners and academics from SA and the United Kingdom (Mannell et al., 2023).

Whilst previous research show links between key health challenges of poor mental health, IPV and HIV, none of the research identified focused on ED when investigating poor mental health. Research on ED in Africa and including South Africa is exceedingly limited despite it potentially being linked to IPV and HIV both which are epidemic in SA. Lack of knowledge about ED can therefore present a missed opportunity in promoting mental health and in the fight against IPV and HIV. Thus this study sought to investigate the impact of SSCF+ on ED among men in rural areas and urban informal settlements in KwaZulu-Natal (KZN), SA.

## Methods

This study used data from a single-blind stratified two-arm cluster pilot randomised controlled trial (RCT) that assessed the efficacy of SSCF+ in reducing IPV, strengthening livelihoods and improving mental health of young people in rural and urban informal settlements in KZN. The research team worked with Project Empower (a local NGO focussed on addressing gender inequalities in KZN) to co-develop the intervention as well as recruit participants and deliver SSCF+.

### Study setting and population

SSCF+ was conducted in urban informal settlements in the eThekwini Municipality and one rural community in Northern KZN, South Africa. In eThekwini Municipality, there are an estimated 587 informal settlements, accounting for approximately one quarter of the eThekwini Municipality population (Misselhorn, 2022). Likewise, approximately 52.5% of KZN’s population of 11.5 million people are reported to live in rural areas (Trade & Investment KwaZulu-Natal, 2020). As discussed earlier, rural areas and urban informal settlements have been reported to have high IPV rates (Allen, 2017; Gibbs et al., 2018), whilst HIV prevalence was found to be high in urban informal settlements and increasing in rural areas (Gibbs et al., 2020). The study areas were also purposefully chosen on the basis that they were safe to conduct the study in, Project Empower assessed the safety of the study areas.

### Intervention

The SSCF+ intervention was adapted from the SSCF intervention (Gibbs et al., 2017). SSCF+ was co-adapted over 18 months with young men and women in the study areas (Mannell et al., 2023). The academics and practitioners supported 17 young people to reflect on the underlying drivers of their experience and perpetration of IPV and develop locally relevant models to explain this and ultimately a theory of change. This theory of change formed the basis for the team to select intervention activities to target mechanisms of change, and these were then tested and refined in conjunction with the young people.

SSCF+ comprised of 15 sessions, each ~3 hours long, primarily delivered to single-sex friendship groups. Sessions were facilitated by same sex trained peer facilitators, who had received 5 weeks of training, with many facilitators coming from communities where the intervention was delivered. Sessions were conducted two times a week. Participants received R100 (~US$5) travel allowance for each session attended.

The intervention focussed on a number of key areas including; addressing mental health drawing on a combination of Self-Help Plus (a stress management course for adults by World Health Organization, 2021) and narrative therapy. Similar to the SSCF, SSCF+ also addressed gender inequalities, challenging the use of violence in relationships and strengthening livelihoods, for instance through encouraging income generating activities and teaching finance management skills.

### Sampling procedures, randomisation, participants and data collection

Given this was a pilot, a power calculation for the known sample size (*N* = 160 male) was conducted, based on pilot data for past 6 months IPV prevalence of approximately 30%. A range of estimates were provided for the power of the sample, showing differences between numbers of clusters, people/cluster and intraclass correlation coefficients, with power ranging from 0.45 to 0.61.

SSCF+ was delivered to clusters which comprised single sex friendship groups. Clusters were formed by group of participants who were recruited via a modified respondent-driven sampling method. Facilitators identified initial young people who met eligibility criteria and asked them to recruit up to 10 of friends who also met the eligibility criteria.

Once clusters were identified (i.e. friendship groups of sizes 5–8), randomisation was conducted using an excel random number generator to allocate clusters in different arms, 1:1 (intervention: delayed control). Randomisation was stratified by communities (i.e. rural and urban informal settlement) and sex (women and men).

The inclusion criteria were; aged 18 to 30 years, a resident in the study clusters and able to communicate in English, isiXhosa and/or isiZulu. The exclusion criteria were; using alcohol or behaviour-altering drugs at the time of recruitment and during any of the research activities, and unable or unwilling to provide informed consent.

**Figure 1.**
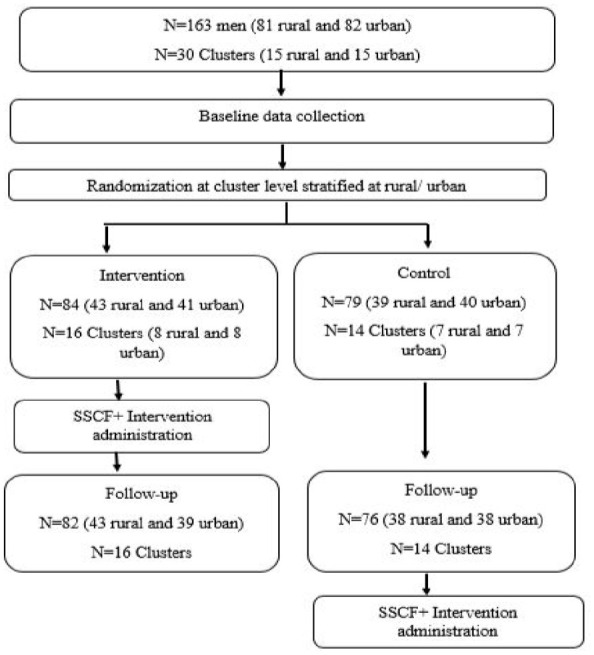
Consort diagram for men enrolled in the SSCF+.

Data collection consisted of two phases, first baseline data (May–June 2023) were collected from the participants prior to randomisation and intervention delivery and the second phase (October-November 2023) approximately 5 months after baseline (about 2–3 months after intervention completion). The control group also received the intervention after end-line data collection. At baseline individuals were blinded to study arms allocation. Data were collected using self-administered questionnaires on a mobile phone, using KoboToolbox with audio support, in either English, isiZulu or isiXhosa, and a trained fieldworker was nearby in case the participants needed help to complete the questionnaire. At end-line participants were tracked using a standardised form and friendships groups. Participants primarily completed the questionnaire in person, but if they could only be contacted via telephone, the fieldworker did a shortened interview telephonically. The consort diagram (Figure 1) summarises information about the enrolment of the men in the SSCF+.

### Ethical consideration

The trial was approved by South Africa Medical Research Council (EC023-10/2022) and the University of Exeter (570602) ethics committees. The trial was pre-registered at clinicaltrials.gov (NCT05783336). The use of data was approved by University of KwaZulu-Natal Biomedical Research Ethics Committee (BREC/00004912/2022). A written informed consent was obtained from all participants.

### Measures

#### Outcome

Emotional dysregulation (ED; Cronbach’s alpha = .91) was the main outcome of the study, measured by the Difficulties in Emotional Dysregulation Scale-16 (DERS-16; Bjureberg et al., 2016) as a valid and reliable scale used in various settings (Fekih-Romdhane et al., 2023; Demirpence Secinti & Sen, 2023; Visted et al., 2023), including in SA (Ward-Smith et al., 2024).We found DERS-16 to be culturally competent to use in urban informal settlements and rural areas in our previous study which is presently under review (Nyoni et al., 2024). DERS-16 has 16 items on how individuals regulate their emotions when they feel upset and the items are split by the five dimensions of ED (i.e. lack of emotional clarity and awareness, inability to engage in goal-oriented behaviours, difficulty controlling impulse behaviours when distressed, lack of confidence in or limited access to effective emotion regulation strategies and none-acceptance of negative emotions). Possible responses were on a 5-point Likert scale (1 = almost never, 2 = sometimes, 3 = about half the time, 4 = most of the time and 5 = almost always). Responses were summed into variable ranging from 16 to 80 and higher scores indicate greater challenges in emotional dysregulation.

#### Exposure variables

As central to the issues facing the youth, the study exposure variables were (1) poverty and other socio-economic challenges and (2) mental health challenges.

##### Poverty related variables

*Food security* (Cronbach’s alpha = .86) was assessed using the Household Hunger Scale (HHS) by Deitchler et al., (2010). The HHS consists of three items asking individuals about challenges in relation to access of food in the past month (e.g. In the past 4 weeks, how often was there was no food to eat of any kind in your house because of a lack of money?), possible responses being, 1 = often, 2 = sometimes, 3 = rarely and 4 = never. A composite variable ranging from 3 to 12 was obtained and recoded into two categories (3–6 as high food insecurity and 7–12 as low food security).

*Stealing for hunger* was assessed when participants were also asked whether they had stolen something as a result of lack of food or money in the past 6 months with responses, ‘never’, ‘once’, ‘2 to 3 times’ and ‘more often’. This was recoded into, ‘never stole in the past 6 months’ and ‘stolen in past 6 months’.

*Employment* was assessed when employment, participants were asked whether they had worked in the past 3 months (‘yes’ or ‘no’).

##### Mental health variables

*Anxiety* (Cronbach’s alpha = .82) was assessed using the Generalised Anxiety Disorder-7 (GAD-7) scale (Spitzer et al., 2006), the responses being, 0 = not at all, 1 = for several days, 2 = over half the days and 3 = nearly every day. The responses were summed to obtain a composite variable that was recoded as two categories where 0 to 10 = no or low symptoms and 11 to 21 = moderate or high symptoms. The GAD-7 has been used in South Africa and in urban informal settlements (Mkhwanazi & Gibbs, 2021).

*Depression* (Cronbach’s alpha = .81) was assessed using the Centre for Epidemiologic Studies Depression Scale (CES-D) with 20 items, having been validated by Radloff (1977) and used in South Africa (Gibbs et al., 2018). The responses on the CES-D were assessed by a 4-point Likert scale, where 0 = rarely or none of the time, 1 = some or a little of the time (1–2 days), 2 = a moderate amount of the time (3–4 days) and 3 = most or all of the time (5–7 days). The responses were summed into a composite variable ranging from 0 to 60 and divided into two categories, where 0 to 20 = no symptoms and 21 to 60 = clinically relevant symptoms of depression (Vilagut et al., 2016).

*Post-traumatic stress* (Cronbach’s alpha = .90) was assessed using the Harvard Trauma Questionnaire (HTQ; Mollica et al., 1992), responses being, 0 = not at all, 1 = a little, 2 = quite a bit and 3 = extremely. Mean scores were calculated for the overall scale and used a cut-off of 2.5 (i.e. a score of 2.4 and below = no symptoms of PTS and 2.5 and above potentially clinically relevant PTS symptoms). The HTQ and a cut-off of 2.5 have been widely used and adopted in SA communities (Machisa et al., 2017; Stoebenau et al., 2023).

Sociodemographic variables that is age, education level and relationship status were also collected.

#### Data analysis

Three analyses were conducted, first summarising sociodemographic and clinical characteristics at baseline using descriptive statistics. We described the sociodemographic and clinical characteristics of the overall sample and compared sociodemographic and clinical characteristics between the intervention and control arms to assess whether randomisation was successful. For categorical variables, we assessed difference using chi-square. For continuous variable, we compared means by study arms based on linear regression. Secondly, we assessed potential baseline sociodemographic and clinical characteristic risk and protective factors for ED at baseline cross-sectionally. We first did bivariate analysis using linear regression and then multivariate analysis. We ran three multiple linear regression models with range of consistent factors (i.e. age, education, relationship, food security, stealing and employment status) and then in each model include one form of mental health (i.e. anxiety, depression and PTS).

To assess our main objective (impact of SSCF+ on ED), we conducted an intention-to-treat (ITT) analysis using generalised estimating equation (GEE) linear regression models. We first fitted unadjusted models and then adjusted models including socio-demographic variables and other baseline descriptors (i.e. age, education, relationship, food security, stealing, employment status and mental health measures). We also undertook supplementary analysis re-fitting similar GEE regression models except the ED outcome measure was dichotomised and the GEE models stratified by risk of depression. Although a widely agreed cut off of the DERS-16 has not yet been established, a cut-off of 39 (16–38 = low ED and 39–80 = high ED) was used based on a study by Burton et al. (2022) among 1049 university students, however, the cut off was based on the risk of depression. Another study similar to ours that conducted a randomised control trial to assess mental health among the youth (Moran et al., 2024), used a DERS-18 cut-off suggested by Burton et al. (2022). We did the supplementary analysis for two reasons. Baneshi and Talei (2011), suggest that dichotomisation might assign participants into an appropriate risk group and improve performance of the models. Other reasons for dichotomisation was to facilitate more understanding and applicability of results for instance allowing expression of differences between treatment and non-treatment groups (Farrington & Loeber, 2000; Purgato & Barbui, 2013). All analyses used STATA 16, and accounting for clustering.

## Results

Of the 163 men who were recruited at baseline, randomisation resulted in 84 (51.53%) from 16 clusters and 79 (48.47%) from 14 clusters being assigned to the intervention and control groups respectively. Only five participants were lost to follow up, resulting in a retention rate of 96.93% (*n* = 158) at end-line.

### Baseline demographics, livelihoods and mental health overall and by intervention arm

Half of the participants (52.76%) were aged 20 to 24 years, and the majority were in some form of intimate relationship (86.88%). Almost half of participants had completed matric/high-school (48.08%). With regard to livelihoods, 60.00% reported having low food security, more than half (68.13%) were unemployed and almost 40.00% admitting having stolen for hunger sometime in the past 6 months. A total of 34.00%, 14.11% and 23.31% of the men were at risk depression, PTS and anxiety respectively. There were no significant differences between the intervention arms (Table 1).

**Table 1.** Overall baseline demographics and by intervention arm.

Variables	Overall	SSCF+	Control	*p*-value
	*N* = 163	*N* = 84	*N* = 79	
Socio-demographics				
Age (years)				
<20	12 (7.36)	5 (5.95)	7 (8.86)	
20–24	86 (52.76)	39 (46.43)	47 (59.49)	
⩾25	65 (39.88)	40 (47.62)	25 (31.65)	.088
Education level				
Primary and secondary	83 (50.92)	39 (46.43)	44 (55.70)	
Matric	80 (49.08)	45 (53.57)	35 (44.30)	.217
Relationship				
In a relationship	139 (86.88)	67 (82.72)	72 (91.14)	
Single	21 (13.12)	14 (17.28)	7 (8.86)	0.118
Livelihoods				
Food security				
Low security	101 (62.35)	52 (62.65)	49 (62.03)	
High security	61 (37.65)	31 (37.35)	30 (37.97)	.934
Stole for hunger				
No	95 (60.13)	46 (58.23)	49 (62.03)	
Yes	63 (39.87)	33 (41.77)	30 (37.97)	.636
Employment				
No	109 (68.12)	56 (69.14)	53 (67.09)	
Yes	51 (31.88)	25 (30.86)	26 (32.91)	.879
Mental health				
Anxiety				
Not at risk	125 (76.69)	65 (77.38)	60 (75.95)	
At risk	38 (23.31)	19 (22.62)	19 (24.05)	.831
Depression				
Not at risk	107 (65.64)	51 (60.71)	56 (70.89)	
At risk	56 (34.36)	33 (39.29)	23 (29.11)	.174
PTS				
Not at risk	140 (85.89)	70 (83.33)	70 (88.61)	
At risk	23 (14.11)	14 (16.67)	9 (11.39)	.303
Emotional dysregulation	*M* (*SD*)	*M* (*SD*)	*M* (*SD*)	*p*-value
	30.01 (10.81)	30.12 (10.36)	29.90 (11.27)	.895

*Note*. ( ) in % unless otherwise specified. PTS = post-traumatic stress.

The mean score for ED was 30.01 (SD=10.81), the minimum and maximum response scores being 15 and 68 respectively. There was no significant difference between arms (Table 1).

### Baseline factors associated with emotional dysregulation

In univariate analysis (Table 2), older age was protective of ED whilst stealing for hunger and all mental health disorders (i.e. depression, PTS and anxiety) were strongly associated with ED. All variables which had a relationship with ED in the univariate analysis remained significant in the multivariate models.

**Table 2.** Bivariate regression analysis of socio-demographics, livelihoods and mental health variables against emotional dysregulation.

Variables	*M*	Unadjusted β [95% CI]	Unadjusted *p*-value
Socio-demographics			
Age (years)			
<20	37.83	Ref	Ref
20–24	29.29	−8.54 [−16.40, −.69]	.033*
⩾25	29.52	−8.31 [−16.05, −.57]	.032*
Education level			
Primary and secondary	30.83	Ref	Ref
Matric	29.16	−1.67 [−5.04, 1.70]	.329
Relationship			
In a relationship	29.86	Ref	Ref
Single	30.86	1.00 [−3.93, 5.93]	.689
Livelihoods			
Food security			
Low security	31.04	Ref	Ref
High security	28.07	−2.97 [−6.22, .27]	.072
Stole for hunger			
No	27.84	Ref	Ref
Yes	33.58	5.75 [2.29, 9.20]	.001*
Employment			
No	30.61	Ref	Ref
Yes	28.65	−1.97 [−5.71, 1.78]	.301
Mental health			
Anxiety			
Not at risk	28.42	Ref	Ref
At risk	35.26	6.85 [2.61, 11.08]	.002*
Depression			
Not at risk	26.19	Ref	Ref
At risk	37.3	11.11 [7.73, 14.49]	<.001*
PTS			
Not at risk	27.74	Ref	Ref
At risk	43.83	16.08 [10.66, 21.50]	<.001*

*Note*. β = coefficient; CI = confidence interval; Ref = reference category; PTS = post-traumatic stress.

* Statistically significant *p* < .05.

After adjusting for possible confounders (Table 3) older age was a protective factor for ED in two of the models (models 1 – anxiety and 3 – PTS). In model 1 (Anxiety), those aged 20 to 24 years (β = −8.80, 95% CI [−17.26, −0.33]) and ⩾25 years (β = −8.91, 95% CI [−17.35, −0.46]) were less likely to have ED. Similarly, in model 3, those aged 20 to 24 years (β = −6.29, 95% CI [−11.36, −1.21]) and ⩾25 years (β = −6.39, 95% CI [−11.52, −1.27]) had lower ED.

**Table 3.** Multivariate regression analysis of socio-demographics, livelihoods and mental health variables against emotional dysregulation.

Variables	Category	Model 1		Model 2		Model 3	
		Adjusted β [95% CI]	Adjusted *p*-value	Adjusted β [95% CI]	Adjusted *p*-value	Adjusted β [95% CI]	Adjusted *p*-value
Age (years)	<20	Ref		Ref		Ref	
	20–24	−8.80 [−17.26, −0.33]	.039*	−6.88 [−15.12, 1.36]	.101	−6.29 [−11.36, −1.21]	.016*
	⩾25	−8.91 [−17.35, −0.46]	.042*	−8.02 [−16.22, 0.18]	.055	−6.39 [−11.52, −1.27]	.015*
Education	Primary and secondary	Ref		Ref		Ref	
	Matric	−1.45 [−4.88, 1.97]	.403	−1.48 [−4.75, 1.79]	.373	−2.39 [−5.28, 0.51]	.106
Relationship	In a relationship	Ref		Ref		Ref	
	Single	−0.88 [−5.57, 3.80]	.71	−1.49 [−5.42, 2.44]	.454	.66 [−3.84, 5.16]	.773
Food security	Low security	Ref		Ref		Ref	
	High security	−0.90 [−4.11, 2.30]	.577	−.40 [−3.11, 2.30]	0.768	−1.29 [−4.06, 1.48]	.357
Stealing	No	Ref		Ref		Ref	
	Yes	4.73 [0.85, 8.61]	.017*	3.53 [0.17, 6.88]	.040*	4.20 [1.02, 7.39]	.010*
Employment	No	Ref		Ref		Ref	
	Yes	−0.86 [−4.39, 2.67]	.63	−1.66 [−4.77, 1.44]	.291	−1.68 [−5.06, 1.71]	.329
Anxiety	Symptoms present	6.71 [2.44, 10.97]	.002*				
Depression	Symptoms present			11.38 [8.06, 14.70]	<.001*		
PTS	Symptoms present					15.85 [10.86, 20.84]	<.001*

β = coefficient; CI = confidence interval; Ref = reference category; PTS = post-traumatic stress.

* Statistically significant *p* < .05.

Men who stole because of hunger were more likely to have increased ED in all the three models. The corresponding adjusted regression coefficients for models 1, 2 and 3 were β = 4.73, 95% CI [0.85, 8.61]), β = 3.53, 95% CI [0.17, 6.88]) and β = 4.20, 95% CI [1.02, 7.39]) respectively (Table 3).

All mental health disorders were strongly correlated with ED after adjusting for confounders. The adjusted regression coefficient of anxiety (model 1) was β = 6.71, 95% CI [2.44, 10.97]), of depression (model 2) was β = 11.38, 95% CI [8.06, 14.70]) and of PTS (model 3) was β = 15.85, (95%CI: [10.86,20.84]; Table 3).

### Impact of Stepping Stones and Creating Futures Plus intervention

SSCF+ did not significantly reduce ED overall (β = −.56, 95% CI [−3.86, 2.74], *p* = .741), adjusted (β = −1.39, 95% CI [−4.09, 1.32], *p* = .314). In models stratified by baseline depression status, SSCF+ significantly reduced ED among participants who were at risk of depression based in both unadjusted models (OR = 0.22, 95% CI [0.07, 0.79]) and adjusted model (aOR 0.12, 95% CI [0.032, 0.46]). ED was not reduced among men not at risk of depression (Table 4).

**Table 4.** Impact of SSCF+ on emotional dysregulation based on generalised estimating equation aggregated by depression.

For those at risk of depression				
	*OR* [95% CI]	*p*-value	*aOR* [95% CI]	*p*-value
SSCF+ intervention	0.22 [0.07, 0.79]	.019*	0.12 [0.03, 0.46]	.002*
For those not at risk of depression				
	*OR* [95% CI]	*p*-value	*aOR* [95% CI]	*p*-value
SSCF+ intervention	0.91 [0.27, 3.06]	.881	0.80 [0.23, 2.75]	.726

*Note. OR* = odds ratio; *aOR* = adjusted odds ratio; CI = confidence interval. The adjusted model was adjusted for all socio-demographics, livelihoods and mental health outcomes.

NB: *Statistically significant *p* < .05.

## Discussion

The study aimed to assess the impact of a pilot randomised control trial (SSCF+) on ED among young men who resided in urban informal settlements and rural areas in KZN, South Africa. The major outcome from our study was that, while SSCF+ did not show a significant reduction in ED in the full sample, it showed a significant reduction of ED among men who were at risk of depression at baseline. We also found that risk factors for ED were age, stealing because of hunger and other measures of poor mental health.

We found SSCF+ to have significantly reduced ED in men at risk of depression, but not in the overall sample possibly due to the strong positive association found between ED and mental health disorders (including depression). It is high likely that men with ED or high ED were concentrated in a sample of those with depressive symptoms compared to the general sample hence the reduction of ED as a result of the intervention being more visible and significant in a sample concentrated with men with ED or high ED.

The positive impact of SSCF+ on ED among men at risk of depression could have occurred through various mechanisms. Firstly, the intervention might have enhanced social connectedness and promoted healthy intimate relationships and both have been reported to be associated with adaptive emotion regulation (which is usually associated with healthier emotion regulation i.e. lowering ED; Marroquín & Nolen-Hoeksema, 2015). Social connectedness might have been enhanced through SSCF+ being delivered in friendship groups and through narrative therapy which the men did in these groups. Narrative therapy enabled the men to freely tell their stories without being judged in these groups, which might have made them feel supported and cared for by their peers in turn enhancing social connectedness. The intervention may also have promoted healthy intimate relationships through challenging use of violence in these relationships. Secondly, the intervention taught basic skills on how to handle stress which is known to be associated with ED (Herts et al., 2012). Thirdly, SSCF+ also reduced poverty, which may have impacted indirectly on ED (Knifton & Inglis, 2020).

In our baseline analysis, we found ED to be associated with poor mental health and stealing for hunger, whilst older age was protective of ED. Similar to our study, all three conditions that is depression, PTS and anxiety have been previously associated with increased ED in individuals (Etkin et al., 2010; Shepherd & Wild, 2014; Visted et al., 2018). Our results suggest that ED is higher or concentrated in people with mental health disorders such as depression, PTS and anxiety, also given the intervention showed a significant reduction of ED in men with depressive symptoms, which supports the notion of it being a trans-diagnostic construct in psychopathology (AKSU et al., 2023; Bierens et al., 2023; Menefee et al., 2022). We also identified stealing because of hunger to be associated with ED, this may be because this measure is a measure of extreme poverty and poverty has a negative impact on one’s mental health (Knifton & Inglis, 2020), poverty also can cause stress in individuals and stress was identified to increase the risk of experiencing ED (Herts et al., 2012). Older age was identified to be protective of ED as reported in other studies (Brummer et al., 2013; Nakagawa et al., 2017). The reason of the former could be due to accumulated knowledge or experience on managing emotions which comes with aging (Charles & Carstensen, 2010). Another study suggested the area responsible for emotions in the brain changes in a way which favours healthy emotion regulation with age (Mather, 2012).

Our study had a number of limitations, the first being that participants were selected through non-probability sampling methods which makes it hard to generalise our findings. Secondly, the sample size was relatively small which might have affected the study’s statistical power. Thirdly, mental health conditions such as depression, PTS and anxiety were measured using screening tools rather than clinically diagnosing participants, though we used well recognised and reliable screening tools. However, we recognise that screeners are not diagnoses and future studies should look at clinically diagnosing participants. Fourth, we could only aggregate our sample by depression since the only identified cut off of DERS-16 during the time of this study was based on depression risk which hindered from also assessing how the intervention would have impacted those at risk of other mental health disorders (i.e. PTS and anxiety).

Notwithstanding the study limitations, the study has various implications. First, although there is conflicting information regarding community based mental health interventions, our findings indicated the SSCF+ may be promising in improving men’s mental health, given the relatively short time of the intervention. Second, to our knowledge, this is the first study of ED among men in urban informal settlements and rural areas in South Africa, finding that it was associated with poor mental health. When planning interventions to address poor mental health, it may be important to consider how to address ED as well. Thirdly, younger men may experience more mental health problems than older men, and may therefore need to be targeted.

## Conclusion

Men who reside in resource limited settings, such as in rural areas and urban informal settlements, who have symptoms of mental health disorders, such as depression, anxiety and PTS, might also be struggling with ED. Community based interventions, such as the SSCF+, which focus on various elements that is improving one’s self-awareness, stress relieving exercises, inter-personal relationship improvement and economic empowerment, show promising results in improving ED among men with symptoms of mental health disorders in these contexts. Thus, with refinement, and the inclusion of issues such as anger management and coping mechanisms to specifically address ED, the approach can be adapted and replicated in similar contexts. Community based interventions could be a turning point in mental health promotion, as they could be a cost-effective way of reaching specific cohorts in a relatively short time, and contribute to the goal of promoting mental health and improving quality of life in communities, particularly those with limited resources. Moreover, improving mental health indirectly contributes to the fight against IPV and HIV, which are both endemic in many resource constrained communities.
